# Kinship analysis on single cells after whole genome amplification

**DOI:** 10.1038/s41598-020-71562-1

**Published:** 2020-09-04

**Authors:** Jana Weymaere, Ann-Sophie Vander Plaetsen, Laurentijn Tilleman, Olivier Tytgat, Kaat Rubben, Sofie Geeraert, Dieter Deforce, Filip Van Nieuwerburgh

**Affiliations:** 1 Laboratory of Pharmaceutical Biotechnology, Ghent University, Ottergemsesteenweg 460, 9000 Gent, Belgium; 2 Department of Life Science Technologies, Imec, 3001 Leuven, Belgium

**Keywords:** Next-generation sequencing, PCR-based techniques, Whole genome amplification

## Abstract

Short Tandem Repeat (STR-) and Single Nucleotide Polymorphism (SNP-) genotyping have been extensively studied within forensic kinship analysis. Nevertheless, no results have been reported on kinship analysis after whole genome amplification (WGA) of single cells. This WGA step is a necessary procedure in several applications, such as cell-based non-invasive prenatal testing (cbNIPT) and pre-implantation genetic diagnosis (PGD). In cbNIPT, all putative fetal cells must be discriminated from maternal cells after enrichment from whole blood. This study investigates the efficacy and evidential value of STR- and SNP-genotyping methods for the discrimination of 24 single cells after WGA, within three families. Formaldehyde-fixed and unfixed cells are assessed in offspring-parent duos and offspring-mother-father trios. Results demonstrate that both genotyping methods can be used in all tested conditions and scenarios with 100% sensitivity and 100% specificity, with a similar evidential value for fixed and unfixed cells. Moreover, sequence-based SNP-genotyping results in a higher evidential value than length-based STR-genotyping after WGA, which is not observed using high-quality offspring bulk DNA samples. Finally, it is also demonstrated that the availability of the DNA genotypes of both parents strongly increases the evidential value of the results.

## Introduction

For many years, length-based Short Tandem Repeat (STR-) genotyping has been the golden standard in paternity testing and forensic kinship analysis^1,2^. However, the benefit of sequence-based Single Nucleotide Polymorphism (SNP-) genotyping combined with STR-genotyping has recently been established in complex kinship cases^3–5^. For example, Mendelian incompatibilities in biologically true paternity cases are less likely to occur using SNP-markers, due to their lower mutation rate compared to STRs, 10^–8^ versus 10^–3^, respectively^6^. This lower mutation rate results in a more stable inheritance of SNPs over generations, which is especially valuable in parentage cases. Furthermore, SNP-markers are more suitable than STR-markers for the analysis of fragmented DNA samples, as the SNP-amplicons are smaller compared to the lengthy STR-amplicons^7^.

Despite the theoretical advantages of SNPs over STRs in forensic casework, there has been much debate whether SNPs will ever fully replace STRs^8^. A considerable disadvantage of SNPs is their less polymorphic nature. Since most SNPs are bi-allelic in the population, 40 or more SNPs are required to provide a similar discriminative power as 13–15 STRs for identification purposes^9–11^. For kinship analysis, even more SNPs are required as shared alleles rather than shared genotypes are examined amongst relatives^11^. Phillips et al*.* stated that on average 60 forensically relevant SNPs are required to match the power of STRs for kinship analysis^11^, while Ayres et al. demonstrated that 50–60 SNPs are required in offspring-mother-father trios, and 70–80 SNPs are required in offspring-parent duos^10^*.*

Due to the emerging interest in SNP-genotyping within forensic casework, the SNP*for*ID consortium was founded in 2003 to establish a PCR-based SNP-genotyping technique with a comparable discriminative power as conventional STR-based methods. Within this consortium, Sanchez et al*.* developed a multiplex PCR assay targeting 52 autosomal SNPs in parallel, further referred to as the ‘SNP*for*ID 52 SNP-plex’^12,13^. Børsting et al*.* demonstrated that this SNP*for*ID panel is a valuable alternative to standard STR-markers in paternity testing^14^.

Most research papers on forensic kinship analysis study the performance of STR- and SNP-based genotyping assays using extracted DNA from either buccal swabs or whole blood as input material^5,15^. However, high-quality DNA is not always available. In some cases, whole genome amplification (WGA) must be performed on low template samples to amplify the input DNA from pg-level up to ng- or µg-level. Unfortunately, WGA is known for its negative influence on the DNA quality by introducing distinct types of errors in the amplified DNA, such as representation bias and sequence errors^16–20^. Consequently, the discrimination of close relatives after WGA might be quite challenging.

Besides in some specific forensic settings^20–23^, discrimination and identification of single cells from close relatives after WGA is also valuable in the field of cell-based non-invasive prenatal testing (cbNIPT). In cbNIPT, the main objective is the isolation of circulating fetal cells from the maternal blood stream to allow genetic analysis of the pure fetal genome in an early stage of gestation^24^. As no unique, specific fetal biomarkers have been discovered yet^25^, enrichment of fetal cells from the pool of maternal blood cells always results in a mixture of fetal and maternal cells. This mixture of cells necessitates identity confirmation of each isolated putative fetal cell prior to downstream genetic analyses, such as copy number variant analysis^26^. Therefore, fetal and maternal cells must be discriminated using STR- or SNP-genotyping after WGA, as WGA is required to allow multiple genetic analyses on a single cell. Based on previous work by our group, comparing different WGA kits^16–18,27^, the SMARTer PicoPLEX Single Cell WGA Kit is preferred for cell lysis and DNA amplification of single cells in a cbNIPT context. This proprietary PicoPLEX technology is based on a linear pre-amplification step using multiple cycles of quasi-random priming, followed by further exponential amplification. The performance of STR- and SNP-genotyping after WGA has been studied profoundly by several research groups^16,18–20^.

Despite the usefulness for both forensic and cbNIPT purposes^18–23^, no research has been done on kinship analysis of close relatives using WGA products of single cells. Therefore, in this study, the efficacy and evidential value of Capillary Electrophoresis (CE-) based STR-genotyping and Next Generation Sequencing (NGS-) based SNP-genotyping were evaluated for the offspring-parent discrimination of 24 single cells after WGA. On the one hand, the commercial AmpFlSTR Identifiler Plus PCR Amplification Kit was preferred in this study for STR-genotyping, as this PCR kit amplifies all thirteen predetermined Combined DNA Index System (CODIS) Core STR-loci, two additional STR-loci^28^, and the gender revealing locus Amelogenin. The benefit of these thirteen CODIS core STR-loci has been recognized worldwide. Not only has the FBI imposed these markers as the golden standard in crime scene investigation in the USA, these core STR-loci are also routinely applied for kinship testing. On the other hand, a multiplex PCR reaction of the 52 SNPs included in the SNP*for*ID 52 SNP-plex was used for SNP-genotyping. Finally, after STR- or SNP-genotyping, the identity of every single cell was determined based on a calculated offspring-parent likelihood ratio (OPLR).

## Materials and methods

### Experimental design

**Figure 1:**
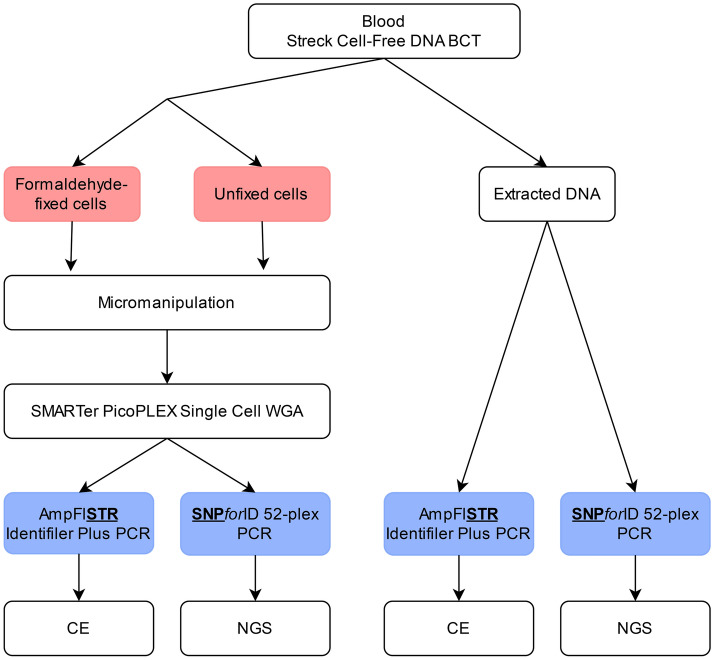
Experimental design.

In this study, the efficacy and evidential value of STR- and SNP-genotyping for the offspring-parent discrimination of single cells after WGA was evaluated. Two scenarios, offspring-parent duos and offspring-mother-father trios, and two conditions, formaldehyde-fixed cells and unfixed cells, were compared. For this purpose, blood was collected from four family members of three families (Fig. 1). 24 single white blood cells, either fixed or not, were obtained via micromanipulation. The DNA of the single cells was then amplified using the SMARTer PicoPLEX Single Cell WGA Kit (Takara Bio Inc., Kusatsu, Japan). All WGA samples were typed once with STR-genotyping and once with SNP-genotyping. Technical repeats after WGA would be of minimal value, as the bias expected from the genotyping step is negligible compared to the expected WGA bias. In addition to single-cell isolation, bulk DNA samples were extracted from the blood samples of all family members to serve as a reference. By comparing the DNA profile of an amplified single cell to the DNA genotype of the reference, an offspring-parent likelihood ratio was calculated. This likelihood ratio was then used to identify each cell, being a parent cell or an offspring cell. Finally, the efficacy and evidential value of the selected STR- and SNP-genotyping methods was evaluated and compared for all given scenarios and conditions.

### Sample collection

Three families were included in the study, each consisting of four family members comprising a mother, a father, and two offspring. All donors were healthy, non-pregnant, and aged over 18 years old. Ethical approval was obtained from the Ghent University Hospital Ethical Committee (B670201942320) and written informed consent was obtained from all participants. All methods were performed in accordance with the ICH Good Clinical Practice rules and the Declaration of Helsinki. From each donor, 9 mL of blood was collected in a Cell-Free DNA BCT blood collection tube (Streck, La Vista, NE, USA). After 12–24 h of preservation at room temperature, bulk DNA samples were extracted using the DNeasy Blood & Tissue kit (Qiagen, Venlo, The Netherlands), quantified with the Qubit dsDNA High Sensitivity Assay kit (ThermoFisher Scientific, Waltham, MA, USA), and stored at − 20 °C until further processing. A Ficoll (GE Healthcare, Chicago, IL, USA) density gradient centrifugation step was performed on the remaining blood, after which the buffy coat was collected. Half of the buffy coat remained unfixed, while the other half was fixed with FIX medium A of the FIX and PERM kit (Invitrogen, Carlsbad, CA, USA), which contains 5–10% formaldehyde. Single fixed and unfixed white blood cells were collected from a serial dilution in phosphate-buffered saline (PBS) (ThermoFisher Scientific, Waltham, MA, USA) via micromanipulation using a STRIPPER pipetter (Origio, Måløv, Denmark) with associated MXL3-IND-100 tips (Origio, Måløv, Denmark). Every single cell was collected in a total volume of 1 µL PBS, snap-frozen in liquid nitrogen, and stored at − 80 °C until further processing.

### Whole genome amplification

Cell lysis and amplification of the micromanipulated single cells was performed with the SMARTer PicoPLEX Single Cell WGA Kit (Takara Bio Inc., Kusatsu, Japan) according to the manufacturer’s recommendations. WGA products were then purified with the Genomic DNA Clean and Concentrator kit (Zymo Research, Irvine, CA, USA) using 5 × ChiP DNA Binding Buffer according to the manufacturer’s instructions. Finally, purified DNA was eluted in 31 µL sterile nuclease-free water at 65 °C, followed by quantification using the Qubit dsDNA High Sensitivity Assay kit (ThermoFisher Scientific, Waltham, MA, USA). A positive control of 1 µL of a 15 pg/µL genomic DNA (Roche, Basel, Switzerland) dilution and a negative control of 1 µL PBS were included during each WGA reaction for quality control.

### STR-genotyping

The WGA products and extracted bulk DNA served as a template for the STR-amplification, using the AmpFlSTR Identifiler Plus PCR amplification kit (ThermoFisher Scientific, Waltham, MA, USA), which is a multiplex PCR kit targeting the gender locus Amelogenin and 15 autosomal STR-loci: D8S1179, D21S11, D7S820, CSF1PO, D3S1358, TH01, D13S317, D16S539, D2S1338, D19S433, VWA, TPOX, D18S51, D5S818, and FGA. For each sample, 10 µL of a 0.1 ng/µL DNA dilution was added to 10 µL AmpFlSTR Identifiler Plus Master Mix and 5 µL AmpFlSTR Identifiler Plus Primer Set. The PCR was performed in a SimpliAmp Thermal Cycler (ThermoFisher Scientific, Waltham, MA, USA) with an initial denaturation step at 95 °C for 11 min, followed by 28 cycles of 94 °C for 20 s and 59 °C for 3 min. A final elongation step at 60 °C for 10 min was performed. A positive control, consisting of 1 ng 2800 M Control DNA (Promega, Madison, WI, USA), and a negative control, consisting of 10 µL sterile nuclease-free water, were included in each STR-PCR reaction for quality control.

STR-profiles were acquired after capillary electrophoresis of the STR-amplicons with the ABI3130xl Genetic Analyzer (ThermoFisher Scientific, Waltham, MA, USA) and analysis with the GeneMapper ID-× 1.2 software (ThermoFisher Scientific, Waltham, MA, USA). An allele (N) is called if the relative fluorescence unit (RFU) is higher than the detection limit of 50 RFU. The N − 1 stutter allele was not called if the RFU is below 30% of the RFU of the N allele. Likewise, the N + 1 allele was not called if the RFU is below 15% of the RFU of the N allele. This correction was applied due to the presence of stutter peaks and the presence of WGA artifacts, respectively. The thresholds were chosen based on previous experiments, as they result in the best STR-profiles after SMARTer PicoPLEX Single Cell WGA Kit, with the lowest number of drop-ins and drop-outs.

### SNP-genotyping

SNP-genotyping was performed on all samples using an in-house optimized multiplex PCR of 52 SNPs, based on the SNP*for*ID 52 SNP-plex protocol developed by the SNP*for*ID consortium^13^. Amplification was performed in 30 µL containing 25 ng WGA product or 10 ng extracted reference DNA, 0.6 U Phusion HotStart II High Fidelity DNA polymerase (Qiagen, Venlo, The Netherlands), 6 nmol dNTPs each (ThermoFisher Scientific, Waltham, MA, USA), 1 × Phusion HF buffer (Qiagen, Venlo, The Netherlands), and 12 µL of a pre-made primer mix. Primer sequences (IDT, Leuven, Belgium) and final primer concentrations can be found in Supplementary Table S1. Amplification was performed in a SimpliAmp Thermal Cycler (ThermoFisher Scientific, Waltham, MA, USA) with an initial denaturation at 98 °C for 30 s, followed by 28 cycles of denaturation at 98 °C for 10 s, annealing at 60 °C for 30 s, and elongation at 72 °C for 30 s. A final elongation step at 72 °C for 10 min was performed.

DNA libraries of the samples were prepared with the NEBNext Ultra II DNA Library Prep Kit for Illumina (New England BioLabs, Ipswich, MA, USA) and NEBNext Multiplex Oligos for Illumina, 96 Unique Dual Index Primer Pairs (New England BioLabs, Ipswich, MA, USA), according to the manufacturer’s recommendations. After library preparation, quantification was performed according to the sequencing library qPCR quantification protocol (Illumina Inc., San Diego, CA, USA) and libraries from all 36 samples were equimolarly pooled to a total concentration of 1.6 nM. Finally, paired-end 150 bp sequencing was performed on a MiSeq sequencer using a MiSeq Reagent Kit v2 Micro (Illumina Inc., San Diego, CA, USA).

The obtained reads (122,000 ± 16,000 reads per sample) were trimmed using cutadapt version 1.16 to remove the Illumina adaptor sequences^29^. Next, the trimmed reads were aligned against the reference sequences of the 52 SNP-loci, consisting of the SNP and 25 nucleotides of flanking region on each side, using Bowtie 2 version 2.2.5 with local alignment settings^30^. Finally, variant calling was performed with SAMtools mpileup and BCFtools version 1.3.1.

A SNP-allele was called if more than 10% of the reads corresponded to that allele and if the total number of reads for that SNP-locus was higher than 10. This implies that a minimum of two reads is required to call an allele. The use of these parameters is necessary to allow for the allele imbalance introduced by the WGA step.

### Evaluation of genotyping quality

For each genotyping method, the locus drop-out rate (LDO %) of all samples was evaluated and compared between different genotyping methods and conditions. The LDO % indicates the percentage of missing loci in the sample profiles. The LDO % was calculated based on Eq. (1).

1 $$LDO \%=\left[1-\left(\frac{total \,number\, of\, loci \,with\, \ge \,1\, observed \,allele}{total\, number \,of\, loci\, in\, the\, multiplex \,PCR} \right)\right]\times 100 \%$$

Likewise, the allele drop-out rate (ADO %) was calculated as shown in Eq. (2). The ADO % indicates the percentage of missing alleles.

2 $$ADO \%=\left[1-\left(\frac{total\, number\, of\, observed \,correct \,alleles}{total \,number\, of \,expected\, alleles \,in \,amplified \,loci} \right)\right]\times 100 \%$$

As a last quality parameter, the percentage of allele drop-ins per locus (allele drop-in rate, DI %) was calculated according to Eq. (3). A called allele was considered as a DI if it was not present in the bulk DNA profile.

3 $$DI \%=\left[1-\left(\frac{total \,number \,of\, observed \,drop-ins}{total \,number \,of\, amplified \,loci} \right)\right]\times 100 \%$$

### Evaluation of efficacy and evidential value

To assess whether the collected single cell is originated from the parent or the offspring, an offspring-parent likelihood ratio (OPLR) was calculated. The OPLR is defined as the ratio of the likelihood that the single cell originates from an offspring of the parent(s) to the likelihood that the single cell originates from the parent, as shown in Eq. (4).

4 $$OPLR = \frac{Likelihood \,that \,the \,single \,cell\, originates\, from\, an \,offspring \,of \,the\, parent(s)}{Likelihood\, that \,the \,single\, cell\, originates\, from \,the\, parent}$$

This OPLR calculation respects allele drop-outs and drop-ins in the DNA profile since WGA often introduces bias. As the occurrence of allele drop-outs and drop-ins is highly variable for the used WGA method between all samples, each likelihood was calculated for different numbers of allele drop-outs and drop-ins. Allele drop-outs and drop-ins varied from 0 to 100% in steps of 5%. For each likelihood calculation, the combination of allele drop-outs and drop-ins with the highest likelihood was selected. This selection method is justifiable, as the percentage of truthful drop-ins and drop-outs will result in the highest likelihood for that relationship. Under- or overestimation of the percentage of drop-ins and drop-outs for a given profile will always result in a lower OPLR. In Supplementary Information S1, all detailed calculations and formulas can be found, based on the method presented by Dørum et al*.*^31^*.* STR-allele frequency data of the Belgian population, determined by the Belgian National Institute for Criminalistics and Criminology, was used for the OPLR calculation after STR-genotyping^32–35^. A frequency of 0.00595 was applied for STR-alleles not included in the Belgian Population database. SNP-allele frequency data of the European population, determined by the SNP*for*ID consortium, was used for the OPLR calculation after SNP-genotyping^36^. A frequency of 10^–12^ was applied for SNP-alleles not included in the SNP-allele frequency database.

Supplementary Figure S1 clarifies all 16 calculated OPLRs per family within this experimental design. A total of 24 OPLR calculations were performed for both the offspring-parent duo scenario and the offspring-mother-father trio scenario. In contrast to offspring-parent duos, for offspring-mother-father trios, the DNA genotype of both parents is available to calculate the likelihood that the cell originates from an offspring of these parents. If the calculated OPLR exceeds 1, the cell was considered as an offspring cell, while if the OPLR is below 1, the cell was considered as a parent cell.

Furthermore, to evaluate the efficacy of both genotyping methods, the sensitivity and specificity were analyzed for the resulting OPLRs of true parent cells and true offspring cells. To allow comparison of the evidential value of the genotyping methods for all conditions and scenarios, the calculated OPLRs were visualized in boxplots, generated with SPSS statistics 26. To define outliers, SPSS uses a step of 1.5 × interquartile range (IQR). Bottom outliers are defined as ${x}_{i}\le {Q}_{1}-1.5\times IQR$, while top outliers are defined as ${x}_{i}{\ge Q}_{3}+1.5\times IQR.$ Finally, to determine the baseline evidential value of the used STR- and SNP-panels, the high-quality bulk DNA samples were also considered in this comparison.

## Results

### Evaluation of genotyping quality

The locus drop-out rate (LDO %) and allele drop-out rate (ADO %) for all samples are calculated for both STR- and SNP-genotyping. All bulk DNA samples result in a LDO % of 0 for both genotyping methods. For single-cell samples, a subdivision between fixed and unfixed cells is made, as shown in Table 1. These results indicate lower drop-out rates for SNP-genotyping than for STR-genotyping after WGA. Besides, SNP-genotyping also shows lower allele drop-in rates (DI %).

**Table 1:** Quality parameters per genotyping method.

	STR-genotyping		SNP-genotyping	
	Unfixed	Fixed	Unfixed	Fixed
Average LDO % 95% CI	17.78 [7.44; 28.12]	15.56 [8.48; 22.63]	5.29 [1.16; 9.42]	8.01 [3.49; 12.53]
Average ADO % 95% CI	19.07 [14.69; 23.45]	15.33 [12.70; 17.96]	10.64 [7.00; 14.29]	13.01 [8.90; 17.11]
DI % 95% CI	11.60 [5.35; 17.85]	15.65 [8.49; 22.81]	0.19 [0; 0.57]	0.18 [0; 0.54]

Since the results show a large spread of the ADO % and DI % between the samples, no fixed value for ADO % and DI % was used in the OPLR calculation. Instead, allele drop-out and drop-in rate varied from 0 to 100% in steps of 5%.

### Efficacy and evidential value of genotyping methods

**Figure 2:**
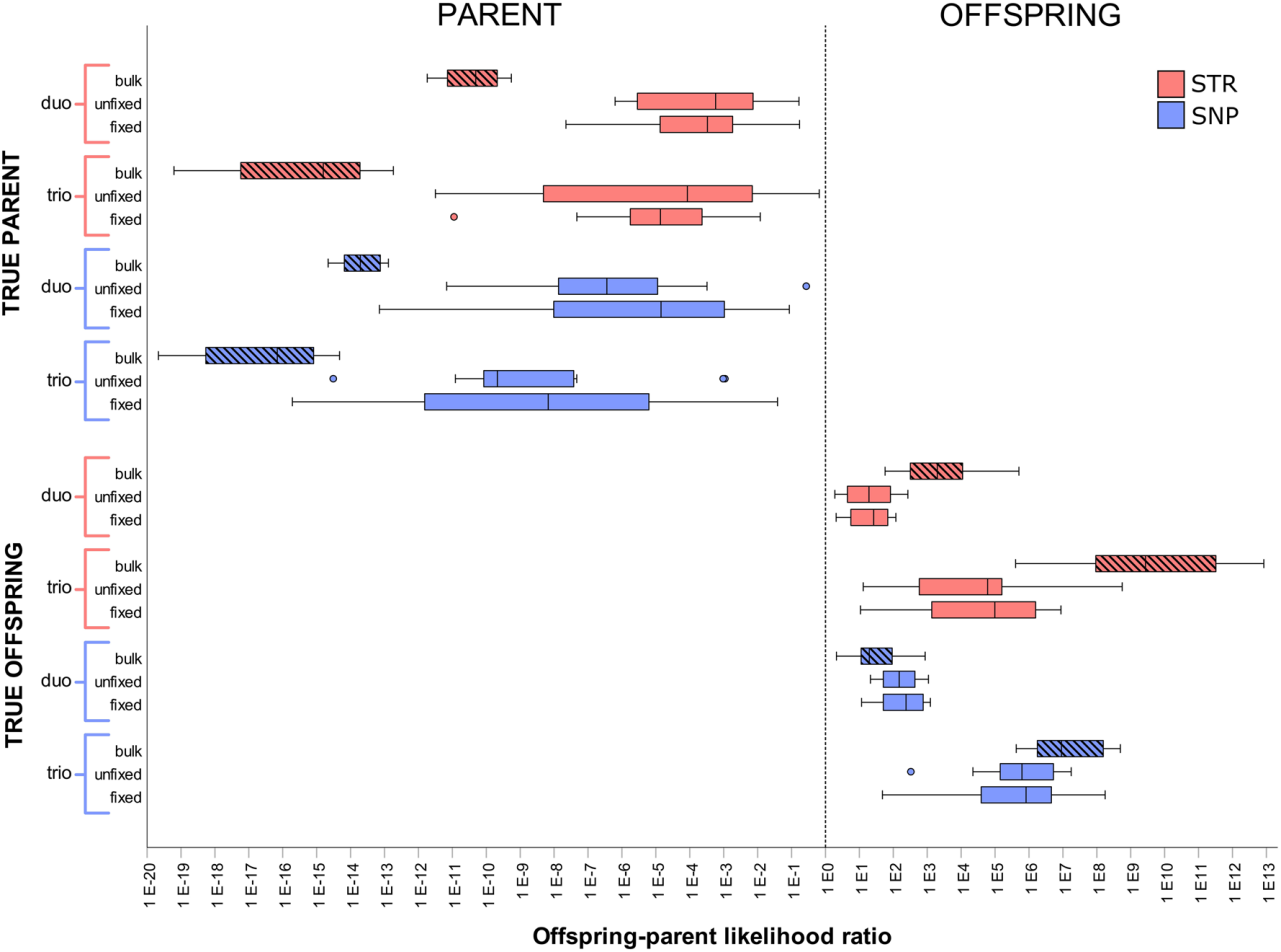
Offspring-parent likelihood ratios (OPLRs) of all single-cell samples and bulk DNA samples per genotyping method, scenario, and condition. A logarithmic scale is used on the x-axis to visualize all OPLR values. Samples with an OPLR below 1 are identified as parents, while samples with an OPLR above 1 are identified as offspring. A larger difference between the OPLR value and 1 implies a higher evidential value of the result. Red indicates the OPLR values for STR-genotyping, while blue indicates the OPLR values for SNP-genotyping. A shaded pattern is used to mark the OPLR boxplots of bulk DNA samples (n = 12), while unfixed (n = 12) and fixed (n = 12) single cells are not shaded. Outliers are illustrated with a small circle.

The offspring-parent likelihood ratio (OPLR) is calculated for all single-cell samples and all bulk DNA samples. Figure 2 shows the OPLRs for STR- and SNP-genotyping, subdivided in duo and trio scenarios. For each scenario, a distinction is made between bulk DNA samples, unfixed cells, and fixed cells. For both true parents and true offspring, the range of OPLRs is visualized by boxplots indicating outliers, minimum, first quartile (Q1), median, third quartile (Q3), and maximum likelihood ratios. The exact values of these boxplots are listed in Supplementary Table S2. Despite notable differences in the median OPLRs, Fig. 2 demonstrates that all methods result in the correct identification of true parent cells (n = 24) and true offspring cells (n = 24) with 100% sensitivity and 100% specificity.

Figure 2 indicates that the identification of true parents and true offspring results in a different level of evidential value. As a larger difference between the median OPLR value and 1 implies a higher evidential value of the result, the evidential value is higher for true parents than for true offspring, both for single-cell samples after WGA and high-quality bulk DNA samples. A worked out example, demonstrating the exact calculation of the OPLR value of a randomly selected true parent and true offspring, is shown in Supplementary Table S3. Furthermore, when comparing duo and trio scenarios within STR- and SNP-genotyping, the evidential value is considerably higher for trios than for duos. For example, for single-cell samples, the evidential value is 10 to 10^3^ times higher in a trio scenario than in a duo scenario.

Next, when comparing the median OPLRs of all true offspring cells, it is noted that the results for unfixed and fixed cells are quite similar within each genotyping method and scenario. However, when unfixed and fixed cells are compared within true parent cells, unfixed cells result in a slightly lower evidential value for STR-genotyping and in a slightly higher evidential value for SNP-genotyping. Therefore, no consistent trend can be determined.

Finally, comparing the median OPLRs of all single-cell samples after SNP-genotyping versus STR-genotyping, SNP-genotyping results in a higher evidential value than STR-genotyping within duo and trio scenarios. For true parent cells, SNP-genotyping results in a 10^2^ to 10^4^ times higher evidential value than STR-genotyping. This higher evidential value of SNP-genotyping versus STR-genotyping is also shown in the parent bulk DNA samples. Likewise, for true offspring cells, the evidential value is 8 times higher for SNP-genotyping than STR-genotyping. However, in offspring bulk DNA samples an inverse trend is noted, indicating a lower evidential value for SNP-genotyping than for STR-genotyping.

## Discussion

The goal of this study was to evaluate the efficacy and evidential value of length-based STR-genotyping and sequence-based SNP-genotyping for the offspring-parent discrimination of single cells after WGA. The suitability of both formaldehyde-fixed and unfixed cells is assessed for this objective in offspring-parent duos and offspring-mother-father trios. Overall, the selected STR- and SNP-based genotyping methods result in the correct identification of single cells with 100% sensitivity and 100% specificity in all tested conditions and scenarios. In this experimental design, conclusions are based on three independent families, which confirms all results in triplicate.

The correct identification of fetal and maternal cells after WGA is extremely valuable in cbNIPT to allow genetic analysis of true fetal cells. The results obtained in this study prove that a higher evidential value is obtained for the identification of true parent cells than for the identification of true offspring cells. This is because the likelihood that the DNA profile of a parent cell is derived from the DNA genotype of this same parent is much higher than the likelihood that the DNA profile of an offspring cell originates from an offspring of the given DNA genotype of the parent. In the latter setting, considerably more uncertainty is introduced, as multiple offspring DNA genotypes can originate from this parent. Therefore, the identification of true parent cells always results in a higher evidential value, both for bulk DNA samples and single cell samples. A worked out example is added in Supplementary Table S3.

In a standard cbNIPT setting, the DNA genotype of the mother is always available. However, results indicate that in a trio scenario, when the DNA genotype of the father is also available, the evidential value of the result is substantially improved. This difference in performance between duo and trio scenarios is also demonstrated for high-quality bulk DNA samples, indicating that this trend is not related to the WGA step. The higher evidential value of trios versus duos can be explained solely by the fact that the presence of both parents excludes all uncertainty of the second parent in the OPLR calculation. The probability that a single cell is an offspring of the given parent(s) is contained in the OPLR calculation. In trios, when the DNA profile of both parents is given, this offspring-parent relationship probability is very high (0.25, 0.5, or 1) or 0, resulting in a more extreme OPLR, while in duos, this probability is much lower as it is based on the allele frequencies in the population. An offspring-mother-father trio scenario is thus always preferred over an offspring-parent duo scenario.

All current cbNIPT workflows consist of multiple enrichment steps to eventually allow the isolation of one or a few fetal cells^24^. Unfortunately, some steps in this workflow also necessitate formaldehyde-fixation of the cells. The negative effect of formaldehyde on the DNA quality is widely recognized but still poorly understood. The available research articles on this subject state that formaldehyde-fixation might hamper downstream PCR amplification and genetic analysis in three ways^37–40^. First, formaldehyde causes DNA-DNA, protein–protein, and DNA–protein crosslinking, which might impede polymerases and inhibit denaturation during PCR amplification. Second, formaldehyde-fixation often results in heavily degraded DNA sequences, which might also impede proper PCR amplification. Third, non-reproducible sequence artifacts can occur due to the deamination of cytosine. Despite these expected negative effects, the results show no consistent trend in evidential value for formaldehyde-fixed and unfixed cells within STR- and SNP-genotyping for duo and trio scenarios. Moreover, as the drop-out rates of STR- and SNP-genotyping are quite similar for fixed and unfixed cells, it appears that the robustness of the SMARTer PicoPLEX Single Cell WGA kit is not critically affected by formaldehyde-fixation. Therefore, in the context of cbNIPT, this WGA kit allows correct discrimination of both fixed and unfixed fetal and maternal single cells with a comparable evidential value.

Finally, when the performance of SNP-genotyping is compared to STR-genotyping within this study objective for single-cell samples, it is demonstrated that SNP-genotyping identifies all single cells with a higher evidential value than STR-genotyping. In true parents, this trend is confirmed by the bulk DNA samples, implying that the SNP*for*ID 52 SNP-plex has a higher discriminative power for identification purposes than the 15 STR-multiplex. However, for the identification of true offspring, bulk DNA samples denote a lower evidential value for SNP-genotyping than for STR-genotyping. Therefore, for kinship analysis, 3.5 SNP-loci per STR-locus appears to be insufficient to reach the same evidential value as STR-genotyping. Nevertheless, true offspring single-cell samples demonstrate a higher evidential value for SNP-genotyping than for STR-genotyping, which is probably caused by the WGA step. According to the manufacturer, after SMARTer PicoPLEX single cell WGA, the length of the amplified DNA fragments is between 100 and 3,000 bp. These shorter fragments might thus impede proper STR-genotyping more than SNP-genotyping, as the SNP-amplicons are smaller compared to the STR-amplicons. This assumption is confirmed by the higher calculated drop-out rate for STR-genotyping than for SNP-genotyping. This higher drop-out rate might also be attributed to a possibly lower sensitivity of CE- over NGS-based allele detection. Either way, after WGA, approximately 12 STR-loci and 48 SNP-loci remain available for the OPLR calculation. This results in 4 SNP-loci per STR-locus, which appears to be sufficient to reach a similar or higher evidential value than STR-genotyping for kinship analysis. Therefore, it can be concluded that SNP-genotyping is always preferred over STR-genotyping after WGA, both in offspring-parent duos and offspring-mother-father trios.

## Conclusion

The selected sequence-based SNP-genotyping and length-based STR-genotyping methods can both be used for offspring versus parent discrimination of single cells in offspring-parent duos and offspring-mother-father trios after WGA with 100% sensitivity and 100% specificity. Moreover, no clear decrease in evidential value is expected after formaldehyde-fixation of the single cells. These findings are valuable in the context of cbNIPT, in which the fetal identity of each isolated putative fetal cell must be confirmed prior to further downstream genetic analyses. Furthermore, the availability of the DNA genotypes of both parents has demonstrated to strongly increase the evidential value of the result. Finally, SNP-genotyping results in a higher evidential value than STR-genotyping within both duo and trio scenarios. Overall, to allow discrimination of single cells with the highest evidential value, sequence-based SNP-genotyping in offspring-mother-father trios is preferred over length-based STR-genotyping.

## Supplementary information

Supplementary information.
