# Supplementary material for: Kinship analysis on single cells after whole genome amplification
Source: Sci Rep. 2020 Sep 4;10:14647. doi: 10.1038/s41598-020-71562-1 (PMC7474072; doi:10.1038/s41598-020-71562-1)
Supplement: Supplementary file 1 — Supplementary information. [file 41598_2020_71562_MOESM1_ESM.pdf]

Kinship analysis on single cells after whole genome amplification

Jana Weymaere[1,#], Ann-Sophie Vander Plaetsen[1,#], Laurentijn Tilleman[1], Olivier Tytgat[1,2], Kaat Rubben[1], Sofie Geeraert[1], Dieter Deforce[1,†], Filip Van Nieuwerburgh[1,†,*]

[1]Laboratory of Pharmaceutical Biotechnology, Ghent University, 9000 Gent, Belgium
[2]Department of Life Science Technologies, Imec, 3001 Leuven, Belgium.
[#] These authors contributed equally.
[*]Corresponding author (email: Filip.VanNieuwerburgh@UGent.be).
[†]These authors jointly supervised.

Contact information:

Prof. Filip Van Nieuwerburgh
Ghent University, Laboratory of Pharmaceutical Biotechnology
Ottergemsesteenweg 460, 9000 Gent, Belgium
email: Filip.VanNieuwerburgh@UGent.be

Supplementary Table S1. Forward and reverse primer sequences and concentrations of the SNP*for*ID primers used in the in-house developed multiplex PCR.

	Primer name, Primer sequence	Final concentration (μM)
1	rs1490413 F, GTGTGGACTGGGCTGATGT<br>rs1490413 R, TTCTCACTAGTGTCCCTGCTCTG	0.5
2	rs876724 F, GCAGGCTCCATTTTTATACCACT<br>rs876724 R, GAATATCTATGAGCAGGCAGTTAGC	0.33
3	rs1357617 F,AGCTGATGCAGACCACTTCAC<br>rs1357617 R, GGATAGCTGATAAGAAACATGACCA	0.5
4	rs2046361 F,CCTATTTGTATGTATCTATTGTCTATGAACG<br>rs2046361 R, GTCATTGTTGACACTTCACCTTCTA	1
5	rs717302 F, CTTTAGAAAGGCATATCGTATTAACTGTG<br>rs717302 R, AACACAGAAAGAGGTTCATATGTTGG	0.33
6	rs1029047 F, CATAACGTGGATTTGTCAGCA<br>rs1029047 R, GGAATAAACTGAAGGCTAAAGAAAAG	1
7	rs917118 F, GCCCTTTAGGGTCGGTTC<br>rs917118 R, GTAAGAGATGACTGAGGTCAACGAG	0.5
8	rs763869 F, ATCAAGTGCTTTCTGTTGACATTTG<br>rs763869 R, GGCTACTCCCTCATAATGTAATGC	1
9	rs1015250 F, AAGTGATGGAGTTAGGAAAAGAACC<br>rs1015250 R, AAGACATTAGGTGGATTCATAGCTG	0.5
10	rs735155 F, GGAGAAAACCGGAGAGCTG<br>rs735155 R, GAGTGTCACCGAATTCAACG	1
11	rs901398 F, CTGGGTGCAAACTAGCTGAATATC<br>rs901398 R, CTGGAATGTACTAGGCAAGAAACTAA	0.33
12	rs2107612 F, GAGCATTCTCTTCTGTTAAAATTGC<br>rs2107612 R, TGAGTACATTATTCAACTGTTTTGGAG	1
13	rs1886510 F, GTCCTTGTCAATCTTTCTACCAGAG<br>rs1886510 R, GGATTTTCACAACAACACTTGC	1
14	rs1454361 F, AGGGAAATACACCCTGAGCTG<br>rs1454361 R, AGCTGTCCATCATCAGTAAGACAC	0.5
15	rs2016276 F, TGCATCCCAGCCTCCACT<br>rs2016276 R, ATTGTACCTTGCCACTTTGTGTG	0.25
16	rs729172 F, CATTAATATGACCAAGGCTCCTCT<br>rs729172 R, ACATTTCCCTCTTGCGGTTAC	0.5
17	rs740910 F, GTATAACAGTTTGCTAAGTAAGGTGAGTG<br>rs740910 R, AGATAGGTTCGAGTTTTGGCTTTA	0.5
18	rs1493232 F, CTATTCTCTCTTTTGGGTGCTAGG<br>rs1493232 R, CAAACTGTTTATTGTGAGGCCTGT	0.5
19	rs719366 F, CCACAGCATCTTTTAACTCTTTTATTATCC<br>rs719366 R, GTAAGGACTTATAGTGAGTAAAGGACAGG	0.75
20	rs1031825 F, CTTATCTTTCCCACATTATGGTCCT<br>rs1031825 R, AAGATATAATCACTGCTTTCAAGTATGC	0.75
21	rs722098 F, GGAAGTACACATCTGTTGACAGTAATGA<br>rs722098 R, GGGTAAAGAAATATTCAGCACATCC	1
22	rs733164 F, AGCTTTCAGCCCCAGGTC<br>rs733164 R, CGGCTCAGGAATGTCAGG	0.5
23	rs826472 F, TGAATTTTGTCTCTGTTATATTAGTCACC<br>rs826472 R, TGTAATTGAAATTTGTAGGCAATAGAC	0.5
24	rs2831700 F, GGCTAAACTATTGCCGGAGA<br>rs2831700 R, TTCCCTAGAACCACAATTATCTGTC	1

	Primer name, Primer sequence	Final concentration (μM)
25	rs873196 F, GCATTCAAATCCCAAGTGCT<br>rs873196 R, GCAGGAGTTGGAGTCAATCAG	0.5
26	rs1382387 F, ACGAAGGAGAAACACCTGAACT<br>rs1382387 R, TGGAGTACTTAATAAGACGCTGCAT	0.5
27	rs2111980 F, AGCATCTTGGCAGCATCC<br>rs2111980 R, AGCAAGATCTTTGCCAGTGAGT	0.5
28	rs2056277 F, CCAAACTGGGTGTTAGGGAGAC<br>rs2056277 R, TCATTATCTCGTCATACTTCCCTGT	0.25
29	rs1024116 F, CCATGTGTTCTAATAAAAAGGATTGC<br>rs1024116 R, TGGGAAGTGAGCAAAAGTAAATACA	0.5
30	rs727811 F, GTGTTTCTTTTTCTCTTACCGGAAC<br>rs727811 R, GTGAATGAAATCATGAGATTGCTG	1
31	rs1413212 F, AACCTCCTTTGGAAACACTGAC<br>rs1413212 R, CAACATTCCATTATCCAGGAGAC	0.5
32	rs938283 F, CATTGAAGTCCTAACCCCTAGTACG<br>rs938283 R, GGATGAGGCCCACCCATA	0.25
33	rs1979255 F, TCAGAGACTATGGATGGTATTTAGGTC<br>rs1979255 R, CATGGAACGTTGGAACTCTTG	0.33
34	rs1463729 F, ACTATCAGTCTCTGCCCTTATTCTG<br>rs1463729 R, CACATGTGCATGCTTTTGG	0.33
35	rs2076848 F, GCCTCACCACCAGAAATCAG<br>rs2076848 R, GACATCAGAAACTCCCATGAAACT	1
36	rs1355366 F, CCATGATTTTCTTGTGGTGAGA<br>rs1355366 R, CACATGTGCTTAGGCCACAAC	0.75
37	rs907100 F, GGAGTTCCTGATAACGATTCTGAAG<br>rs907100 R, ACAGAAAAGAAGCCCAGTTGGA	0.5
38	rs354439 F, GGCTTCTCTTTCCCTTATGTATCTC<br>rs354439 R, CAGGTTGCGATAGAAAACAGTGAAT	0.25

	Primer name, Primer sequence	Final concentration (μM)
39	rs2040411 F, TCTGGAATGCCAGTTCTTTTGT<br>rs2040411 R, CAGAACGCCTATGAAAACCAGT	0.33
40	rs737681 F, ACATGTGAGGCCATCTCCAC<br>rs737681 R, CCTTACTGTGATGTAGGCACTGTTC	0.75
41	rs2830795 F, CACTTCTATAGACATAGGACACACCAT<br>rs2830795 R, ATCTAGGCTCTGAATCAGGATGAG	0.25
42	rs251934 F, AGAGGGCAGTGAGGCTTTTAAGTAG<br>rs251934 R, TGCTAGAATCCAGACTTAACTACCAG	0.25
43	rs914165 F, AGCAGCAGAGCCTGGATG<br>rs914165 R, AGACCAGTCACCTCTTTTGCACT	1
44	rs10495407 F, AGATCTCCACTTCCTCTTGGTTG<br>rs10495407 R, CTCCCAAATTTACATTGCCACT	0.33
45	rs1360288 F, AGACTCTCTGTGTGTGGCTTTG<br>rs1360288 R, GAGGGGGCATCTGTTGAG	1
46	rs964681 F, GTACCTGGAGGTGATTTCTGTGAG<br>rs964681 R, GTTATGGAGGATTGGTAAGAACCAG	0.5
47	rs1005533 F, GGTTTGTGTGTGAGTGTTTCAGAT<br>rs1005533 R, CCTTATGCCTCCCCTGAAC	0.5
48	rs8037429 F, TTCACTTTGCTACACCTCCATAGTA<br>rs8037429 R, TGCTACGTAAGAGGTCATTGCTATC	0.33
49	rs891700 F, TTTTCAGAGGTGGTATTTCTAGCTG<br>rs891700 R, GCTATGACACTCCTTAGAACTATGCAA	0.75
50	rs1335873 F, GTGGATGATATGGTTTCTCAAGG<br>rs1335873 R, TTCAACAAACGTGTGATGCTCT	0.33
51	rs1028528 F, ACAGCTGATGCCTCCCTGA<br>rs1028528 R, GAGGATGAAGGTTAGAGCCAGAC	0.33
52	rs1528460 F, TCCTGGAGATCAATATTTAGCCTTA<br>rs1528460 R, GGGTGACCAGTAGTTCTATGAGC	1

<u>Supplementary Information S1.</u> Offspring-parent likelihood ratio (OPLR) calculation for offspring-parent duos (A) and offspring-parents trios (B).

A. OPLR calculation for offspring-parent duos

$$OPLR = \frac{Likelihood\ that\ the\ cell\ originates\ from\ an\ offspring\ of\ the\ parent}{Likelihood\ that\ the\ cell\ originates\ from\ the\ parent}$$

$$= \prod_{l \in L} \frac{\sum_{u \in U} P(\mathcal{M}_u = M | g_o = u, d, c) \cdot P(g_o = u | H_{op})}{P(\mathcal{M}_p = M | g_p, d, c)}$$

$$= \prod_{l \in L} \frac{\sum_{u \in U} \left[\prod_{a \notin M} P(a \notin \mathcal{M}_u | g_o = u, d, c) \cdot \prod_{a \in M} P(a \in \mathcal{M}_u | g_o = u, d, c) \cdot P(g_o = u | H_{op})\right]}{\prod_{a \notin M} P(a \notin \mathcal{M}_p | g_p, d, c) \cdot \prod_{a \in M} P(a \in \mathcal{M}_p | g_p, d, c)}$$

$$= \prod_{l \in L} \frac{\sum_{u \in U} \left[\prod_{a \notin M} [(1 - cf_a) \cdot d^{n_{a_u}}] \cdot \prod_{a \in M} [(1 - (1 - cf_a)) \cdot d^{n_{a_u}}] \cdot P(g_o = u | H_{op})\right]}{\prod_{a \notin M} [(1 - cf_a) \cdot d^{n_{a_p}}] \cdot \prod_{a \in M} [(1 - (1 - cf_a)) \cdot d^{n_{a_p}}]}$$

$$\mathrm{g_p} = (a_{p_1}, a_{p_2})$$

$$u = (a_{u_1}, a_{u_2})$$

$$P(g_o = u | H_{op}) \begin{cases} \text{if } a_{p_1} = a_{p_2} \begin{cases} f_{a_{u_1}}, \text{if } a_{u_1} = a_{u_2} \text{ and } a_{u_1} = a_{p_1} \\ f_{a_{u_1}}, \text{if } a_{u_1} \neq a_{u_2} \text{ and } a_{u_2} = a_{p_1} \\ f_{a_{u_2}}, \text{if } a_{u_1} \neq a_{u_2} \text{ and } a_{u_1} = a_{p_1} \\ 0, \quad otherwise \end{cases} \\ \text{if } a_{p_1} \neq a_{p_2} \begin{cases} 0.5 \cdot f_{a_{u_1}}, \text{if } a_{u_1} = a_{u_2} \text{ and } (a_{u_1} = a_{p_1} \text{ or } a_{u_1} = a_{p_2}) \\ 0.5 \cdot f_{a_{u_1}} + 0.5 \cdot f_{a_{u_2}}, \text{if } a_{u_1} \neq a_{u_2} \text{ and } (a_{u_1} = a_{p_1} \text{ and } a_{u_2} = a_{p_2}) \\ 0.5 \cdot f_{a_{u_1}} + 0.5 \cdot f_{a_{u_2}}, \text{if } a_{u_1} \neq a_{u_2} \text{ and } (a_{u_1} = a_{p_2} \text{ and } a_{u_2} = a_{p_1}) \\ 0.5 \cdot f_{a_{u_1}}, \text{if } a_{u_1} \neq a_{u_2} \text{ and } (a_{u_2} = a_{p_1} \text{ and } a_{u_1} \neq a_{p_2}) \\ 0.5 \cdot f_{a_{u_1}}, \text{if } a_{u_1} \neq a_{u_2} \text{ and } (a_{u_2} = a_{p_2} \text{ and } a_{u_1} \neq a_{p_1}) \\ 0.5 \cdot f_{a_{u_2}}, \text{if } a_{u_1} \neq a_{u_2} \text{ and } (a_{u_1} = a_{p_1} \text{ and } a_{u_2} \neq a_{p_2}) \\ 0.5 \cdot f_{a_{u_2}}, \text{if } a_{u_1} \neq a_{u_2} \text{ and } (a_{u_1} = a_{p_2} \text{ and } a_{u_2} \neq a_{p_1}) \\ 0, \quad otherwise \end{cases} \end{cases}$$

Whereby l is one locus in the set of all tested loci L, $u = (a_{u_1}, a_{u_2})$ is a genotype in the set of all possible genotypes of an offspring U, a is an allele in the locus l, $g_o = (a_{o_1}, a_{o_2})$ is the unknown genotype of the offspring, $\mathcal{M}_u$ is a random variable representing the DNA profile of the offspring, $\mathcal{M}_p$ is a random variable representing the DNA profile of the parent, M is the observed mixture of alleles for locus l, $d = ADO$ % is the allele drop-out rate, $c = DI$ % is the percentage of allele drop-ins per locus, $g_p = (a_{p_1}, a_{p_2})$ is the known genotype of the parent, $n_a = \{0,1,2\}$ denotes the number of times allele a is observed in the genotype of the offspring u or the reference parent p, H_{op} specifies the offspring-parent relationship, and f_a is the frequency of allele a in the population.

B. OPLR calculation for offspring-mother-father trios

$$OPLR = \frac{Likelihood\ that\ the\ cell\ originates\ from\ an\ offspring\ of\ the\ parents}{Likelihood\ that\ the\ cell\ originates\ from\ the\ parent}$$

$$= \prod_{l \in L} \frac{\sum_{u \in U} P(\mathcal{M}_u = M | g_o = u, d, c) \cdot P(g_o = u | H_{op})}{P(\mathcal{M}_{p_1} = M | g_{p_1}, d, c)}$$

$$= \prod_{l \in L} \frac{\sum_{u \in U} \left[\prod_{a \notin M} P(a \notin \mathcal{M}_u | g_o = u, d, c) \cdot \prod_{a \in M} P(a \in \mathcal{M}_u | g_o = u, d, c) \cdot P(g_o = u | H_{op})\right]}{\prod_{a \notin M} P(a \notin \mathcal{M}_{p_1} | g_{p_1}, d, c) \cdot \prod_{a \in M} P(a \in \mathcal{M}_{p_1} | g_{p_1}, d, c)}$$

$$= \prod_{l \in L} \frac{\sum_{u \in U} \left[\prod_{a \notin M} [(1 - cf_a) \cdot d^{n_{a_u}}] \cdot \prod_{a \in M} [(1 - (1 - cf_a)) \cdot d^{n_{a_u}}] \cdot P(g_o = u | H_{op})\right]}{\prod_{a \notin M} [(1 - cf_a) \cdot d^{n_{a_{p_1}}}] \cdot \prod_{a \in M} [(1 - (1 - cf_a)) \cdot d^{n_{a_{p_1}}}]}$$

$$g_{p_1} = (a_{p_{1,1}}, a_{p_{1,2}})$$

$$g_{p_2} = (a_{p_{2,1}}, a_{p_{2,2}})$$

$$u = (a_{u_1}, a_{u_2})$$

$$
P\left(g_u = u \middle| H_{op}\right)
\begin{cases}
\text{if } a_{p_{1,1}} = a_{p_{1,2}} = a_{p_{2,1}} = a_{p_{2,2}}
\begin{cases}
1, \text{ if } a_{u_1} = a_{u_2} \text{ and } a_{u_1} = a_{p_{1,1}} \\
0,\ \textit{otherwise}
\end{cases} \\
\text{if } a_{p_{1,1}} = a_{p_{1,2}}\ \textit{and}\ a_{p_{2,1}} \neq a_{p_{2,2}}
\begin{cases}
\text{if } a_{p_{1,1}} = a_{p_{2,1}}
\begin{cases}
0.5, \text{ if } a_{u_1} = a_{u_2} \text{ and } a_{u_1} = a_{p_{1,1}} \\
0.5, \text{ if } a_{u_1} = a_{p_{1,1}} \text{ and } a_{u_2} = a_{p_{2,2}} \\
0.5, \text{ if } a_{u_1} = a_{p_{2,2}} \text{ and } a_{u_2} = a_{p_{1,1}} \\
0, \textit{otherwise}
\end{cases} \\
\text{if } a_{p_{1,1}} = a_{p_{2,2}}
\begin{cases}
0.5, \text{ if } a_{u_1} = a_{u_2} \text{ and } a_{u_1} = a_{p_{1,1}} \\
0.5, \text{ if } a_{u_1} = a_{p_{1,1}} \text{ and } a_{u_2} = a_{p_{2,1}} \\
0.5, \text{ if } a_{u_1} = a_{p_{2,1}} \text{ and } a_{u_2} = a_{p_{1,1}} \\
0, \textit{otherwise}
\end{cases} \\
\textit{otherwise}
\begin{cases}
0.5, \text{ if } a_{u_1} = a_{p_{1,1}} \text{ and } a_{u_2} = a_{p_{2,1}} \\
0.5, \text{ if } a_{u_1} = a_{p_{1,1}} \text{ and } a_{u_2} = a_{p_{2,2}} \\
0.5, \text{ if } a_{u_1} = a_{p_{2,1}} \text{ and } a_{u_2} = a_{p_{1,1}} \\
0.5, \text{ if } a_{u_1} = a_{p_{2,2}} \text{ and } a_{u_2} = a_{p_{1,1}} \\
0, \textit{otherwise}
\end{cases}
\end{cases} \\
\text{if } a_{p_{1,1}} \neq a_{p_{1,2}}\ \textit{and}\ a_{p_{2,1}} = a_{p_{2,2}}
\begin{cases}
\text{if } a_{p_{1,1}} = a_{p_{2,1}}
\begin{cases}
0.5, \text{ if } a_{u_1} = a_{u_2} \text{ and } a_{u_1} = a_{p_{1,1}} \\
0.5, \text{ if } a_{u_1} = a_{p_{1,1}} \text{ and } a_{u_2} = a_{p_{1,2}} \\
0.5, \text{ if } a_{u_1} = a_{p_{1,2}} \text{ and } a_{u_2} = a_{p_{1,1}} \\
0, \textit{otherwise}
\end{cases} \\
\text{if } a_{p_{1,2}} = a_{p_{2,1}}
\begin{cases}
0.5, \text{ if } a_{u_1} = a_{u_2} \text{ and } a_{u_1} = a_{p_{1,2}} \\
0.5, \text{ if } a_{u_1} = a_{p_{1,2}} \text{ and } a_{u_2} = a_{p_{1,1}} \\
0.5, \text{ if } a_{u_1} = a_{p_{1,1}} \text{ and } a_{u_2} = a_{p_{1,2}} \\
0, \textit{otherwise}
\end{cases} \\
\textit{otherwise}
\begin{cases}
0.5, \text{ if } a_{u_1} = a_{p_{1,1}} \text{ and } a_{u_2} = a_{p_{2,1}} \\
0.5, \text{ if } a_{u_1} = a_{p_{1,2}} \text{ and } a_{u_2} = a_{p_{2,1}} \\
0.5, \text{ if } a_{u_1} = a_{p_{2,1}} \text{ and } a_{u_2} = a_{p_{1,1}} \\
0.5, \text{ if } a_{u_1} = a_{p_{2,1}} \text{ and } a_{u_2} = a_{p_{1,2}} \\
0, \textit{otherwise}
\end{cases}
\end{cases} \\
\text{if } a_{p_{1,1}} \neq a_{p_{1,2}}\ \textit{and}\ a_{p_{2,1}} \neq a_{p_{2,2}}
\begin{cases}
\text{if } a_{p_{1,1}} = a_{p_{2,1}}\ \textit{and}\ a_{p_{1,2}} = a_{p_{2,2}}
\begin{cases}
0.25, \text{ if } a_{u_1} = a_{u_2} = a_{p_{1,1}} \\
0.25, \text{ if } a_{u_1} = a_{u_2} = a_{p_{1,2}} \\
0.5, \text{ if } a_{u_1} = a_{p_{1,1}} \text{ and } a_{u_2} = a_{p_{1,2}} \\
0.5, \text{ if } a_{u_1} = a_{p_{1,2}} \text{ and } a_{u_2} = a_{p_{1,1}} \\
0, \textit{otherwise}
\end{cases} \\
\text{if } a_{p_{1,2}} = a_{p_{2,1}}\ \textit{and}\ a_{p_{1,1}} = a_{p_{2,2}}
\begin{cases}
0.25, \text{ if } a_{u_1} = a_{u_2} = a_{p_{1,1}} \\
0.25, \text{ if } a_{u_1} = a_{u_2} = a_{p_{1,2}} \\
0.5, \text{ if } a_{u_1} = a_{p_{1,1}} \text{ and } a_{u_2} = a_{p_{1,2}} \\
0.5, \text{ if } a_{u_1} = a_{p_{1,2}} \text{ and } a_{u_2} = a_{p_{1,1}} \\
0, \textit{otherwise}
\end{cases} \\
\textit{otherwise}
\begin{cases}
0.25, \text{ if } a_{u_1} = a_{p_{1,1}} \text{ and } a_{u_2} = a_{p_{2,1}} \\
0.25, \text{ if } a_{u_1} = a_{p_{1,1}} \text{ and } a_{u_2} = a_{p_{2,2}} \\
0.25, \text{ if } a_{u_1} = a_{p_{1,2}} \text{ and } a_{u_2} = a_{p_{2,1}} \\
0.25, \text{ if } a_{u_1} = a_{p_{1,2}} \text{ and } a_{u_2} = a_{p_{2,2}} \\
0.25, \text{ if } a_{u_1} = a_{p_{2,1}} \text{ and } a_{u_2} = a_{p_{1,1}} \\
0.25, \text{ if } a_{u_1} = a_{p_{2,2}} \text{ and } a_{u_2} = a_{p_{1,1}} \\
0.25, \text{ if } a_{u_1} = a_{p_{2,1}} \text{ and } a_{u_2} = a_{p_{1,2}} \\
0.25, \text{ if } a_{u_1} = a_{p_{2,2}} \text{ and } a_{u_2} = a_{p_{1,2}} \\
0, \textit{otherwise}
\end{cases}
\end{cases}
\end{cases}
$$

Whereby l is one locus in the set of all tested loci L, $u = (a_{u_1}, a_{u_2})$ is a genotype in the set of all possible genotypes of an offspring U, a is an allele in the locus l, $g_o = (a_{o_1}, a_{o_2})$ is the unknown genotype of the offspring, $\mathcal{M}_u$ is a random variable representing the DNA profile of the offspring, $\mathcal{M}_{p_1}$ is a random variable representing the DNA profile of the reference parent, M is the observed mixture of alleles for locus l, $d = ADO$ % is the allele drop-out rate, $c = DI$ % is the percentage of allele drop-ins per locus, $g_{p_1} = (a_{p_{1,1}}, a_{p_{1,2}})$ is the known genotype of the reference parent, $g_{p_2} = (a_{p_{2,1}}, a_{p_{2,2}})$ is the known genotype of the second parent, $n_a = \{0,1,2\}$ denotes the number of times allele a is observed in the genotype of the offspring u or the reference parent p_1, H_{op} specifies the offspring-mother-father relationship, and f_a is the frequency of allele a in the population.

<u>Supplementary Figure S1.</u> All 16 calculated OPLRs per family within this experimental design. **A.** OPLR calculation of true offspring cells. **B.** OPLR calculation of true parent cells.

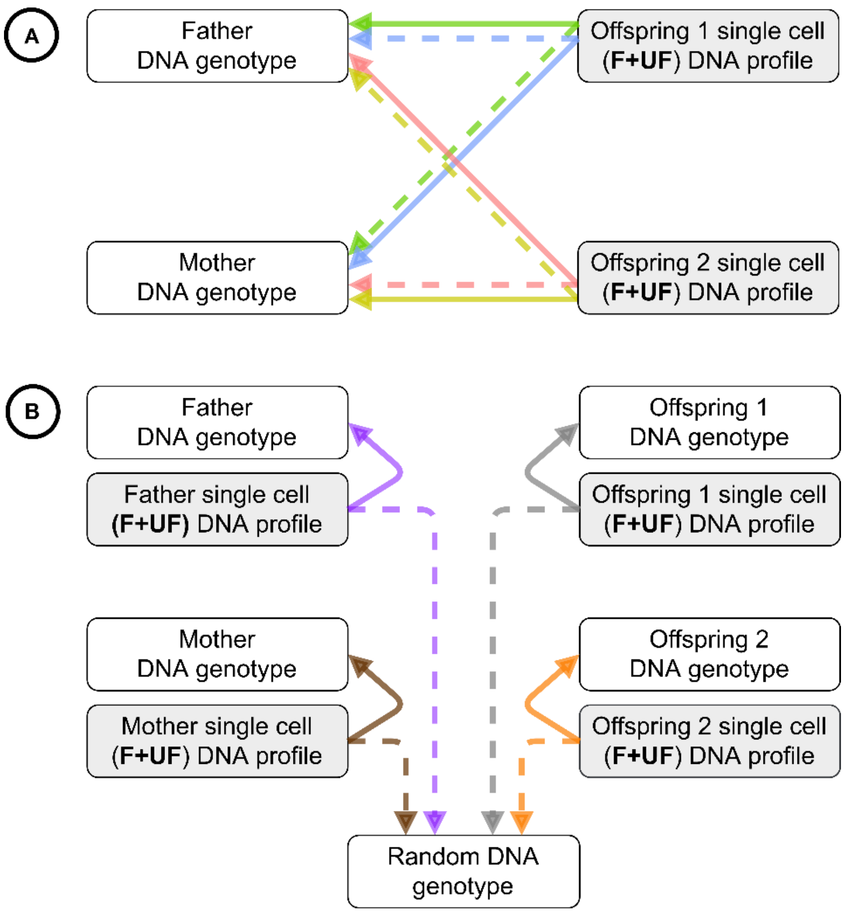

Figure A indicates the OPLR calculation of true offspring cells. The arrows connect the DNA profile of a single cell to the DNA genotype to which it is compared in an OPLR calculation. Each color represents two calculated OPLRs: the full line is considered in the OPLR calculation of a duo scenario, while both the full line and the dotted line are considered in the OPLR calculation of a trio scenario.

Figure B indicates the OPLR calculation of true parent cells, using the same color and line code as figure A. To augment the sample number, the DNA profiles of biological offspring cells are also used in the OPLR calculation of true parent cells, by comparing it to its own DNA genotype. A random unrelated DNA genotype was used in the OPLR calculation of the trio scenarios, representing the real situation in which both parents are supposed to be unrelated.

<u>Supplementary Table S2.</u> Minimum, first quartile (Q1), median, third quartile (Q3), and maximum OPLR per situation as shown in the boxplots for all single-cell samples (fixed and unfixed) and all bulk DNA samples.

		STR-genotyping						SNP-genotyping					
		DUO			TRIO			DUO			TRIO		
		Bulk DNA	Unfixed	Fixed	Bulk DNA	Unfixed	Fixed	Bulk DNA	Unfixed	Fixed	Bulk DNA	Unfixed	Fixed
TRUE PARENTS	Min	1.8E-12	6.3E-07	2.2E-08	6.2E-20	3.2E-12	4.7E-08	2.2E-15	6.8E-12	7.1E-14	2.2E-20	1.2E-11	1.9E-16
	Q1	6.9E-12	2.5E-06	1.2E-05	5.1E-18	8.2E-09	1.6E-06	6.1E-15	1.2E-08	9.4E-09	5.1E-19	7.4E-11	4.8E-12
	Med	6.4E-11	5.8E-04	3.3E-04	1.7E-15	1.0E-04	1.5E-05	2.1E-14	3.9E-07	1.7E-05	7.0E-17	2.1E-10	7.6E-09
	Q3	2.3E-10	1.6E-02	1.9E-03	2.4E-14	5.2E-02	3.1E-04	7.5E-14	1.2E-05	1.2E-03	2.0E-15	4.3E-08	8.2E-06
	Max	5.5E-10	1.7E-01	1.7E-01	1.8E-13	6.6E-01	1.2E-02	1.3E-13	3.2E-04	8.6E-02	4.7E-15	4.7E-08	3.9E-02
TRUE OFFSPRING	Min	5.7E+01	1.9E+00	2.1E+00	4.0E+05	1.3E+01	1.1E+01	2.1E+00	2.1E+01	1.2E+01	4.2E+05	2.2E+04	4.8E+01
	Q1	5.3E+02	3.9E+00	5.2E+00	8.1E+07	5.1E+02	1.2E+03	1.1E+01	4.9E+01	5.0E+01	1.5E+06	1.2E+05	3.8E+04
	Med	2.7E+03	1.9E+01	2.7E+01	3.0E+09	6.9E+04	1.2E+05	2.0E+01	1.5E+02	2.4E+02	9.3E+06	6.2E+05	8.3E+05
	Q3	1.1E+04	1.1E+02	7.5E+01	4.7E+11	1.7E+05	2.7E+06	1.0E+02	5.3E+02	8.2E+02	2.7E+08	8.2E+06	4.8E+06
	Max	5.1E+05	2.7E+02	1.2E+02	8.4E+12	5.6E+08	8.8E+06	8.7E+02	1.1E+03	1.2E+03	5.0E+08	1.8E+07	1.8E+08

Supplementary Table S3. Worked out example of an OPLR calculation for parent bulk, offspring bulk, a true parent cell, and a true offspring cell.

STR	Parent bulk vs. Parent bulk		Offspring bulk vs. Parent bulk		Parent cell vs. Parent bulk		Offspring cell vs. Parent bulk	
	OFFSPRING LIKELIHOOD	PARENT LIKELIHOOD	OFFSPRING LIKELIHOOD	PARENT LIKELIHOOD	OFFSPRING LIKELIHOOD	PARENT LIKELIHOOD	OFFSPRING LIKELIHOOD	PARENT LIKELIHOOD
DI% with highest likelihood	0	0	15	95	0	20	25	95
ADO% with highest likelihood	0	0	5	50	10	20	10	55
D3S1358	1.43E-01	1.00E+00	1.05E-01	3.47E-02	1.18E-01	1.35E-01	9.24E-02	3.52E-02
D5S818	1.70E-01	1.00E+00	5.47E-05	4.69E-04	2.87E-01	6.17E-02	1.63E-04	5.67E-04
D13S317	9.76E-02	1.00E+00	2.53E-01	1.17E-01	1.78E-01	7.99E-01	2.20E-01	1.10E-01
D19S433	2.26E-01	1.00E+00	7.33E-02	2.02E-02	9.18E-02	1.33E-01	1.83E-04	1.16E-04
TPOX	3.97E-01	1.00E+00	3.70E-01	3.82E-01	3.06E-01	1.49E-01	2.72E-01	2.42E-01
D21S11	1.24E-01	1.00E+00	8.90E-02	2.79E-02	1.00E-01	5.47E-01	7.78E-02	2.84E-02
D16S539	3.07E-01	1.00E+00	5.59E-02	2.08E-02	2.49E-01	6.10E-01	5.05E-02	2.16E-02
D18S51	1.41E-01	1.00E+00	5.03E-02	1.49E-02	1.14E-01	5.59E-01	4.35E-02	1.51E-02
D7S820	1.68E-01	1.00E+00	3.35E-02	1.32E-02	2.41E-01	8.11E-01	2.89E-02	1.24E-02
CSF1PO	3.48E-01	1.00E+00	2.70E-01	1.60E-01	4.04E-01	8.41E-01	2.48E-01	1.54E-01
TH01	2.02E-01	1.00E+00	6.17E-02	1.98E-02	1.39E-01	1.37E-01	1.08E-02	1.67E-02
vWA	2.46E-01	1.00E+00	4.25E-02	1.38E-02	1.99E-01	5.91E-01	3.75E-02	1.41E-02
FGA	1.16E-01	1.00E+00	9.46E-02	1.38E-01	9.40E-02	5.52E-01	8.06E-02	1.16E-01
D8S1179	1.65E-01	1.00E+00	3.16E-02	8.32E-03	2.84E-02	1.97E-03	1.21E-01	1.41E-01
D2S1338	9.12E-02	1.00E+00	6.70E-02	1.10E-01	7.39E-02	5.46E-01	7.80E-02	1.11E-01
likelihood	5.85E-12	1	4.70E-20	9.23E-24	2.58E-13	8.03E-10	6.19E-23	4.67E-25
OPLR	5.85E-12		5.09E+03		3.21E-04		1.33E+02	

For this worked out example, a parent STR-profile and an offspring STR-profile were randomly selected to demonstrate the exact likelihoods and OPLR values. Bulk and single cell samples are always compared to the related parent bulk DNA-profile. For each sample, the offspring likelihood and the parent likelihood is calculated per locus. By multiplying all individual likelihoods, a final offspring likelihood and a final parent likelihood is obtained. The fraction of the offspring likelihood divided by the parent likelihood results in the OPLR.

This table demonstrates that more extreme OPLR values and thus higher evidential values are calculated for true parent samples than for true offspring samples. For true parent bulk samples, the parent likelihood is equal to 1, whereas for true offspring bulk samples the offspring likelihood will always remain below 1. In the latter setting, much more uncertainty is introduced as multiple offspring DNA genotypes can originate from the parent. Therefore, both for bulk and single cell samples, the OPLR values for true offspring will always be less extreme than for true parents resulting in a lower evidential value.
